# Tiny? Make it mighty! Maximizing a limited-budget upgrade of a pint-sized hospital library using UX methods

**DOI:** 10.29173/jchla29774

**Published:** 2024-12-01

**Authors:** Sarah Visintini, Jessica McEwan

**Affiliations:** 1 Berkman Librarian, University of Ottawa Heart Institute and University of Ottawa, Ottawa, ON; 2 User Experience Librarian, University of Ottawa, Ottawa, ON

## Abstract

**Introduction:**

The University of Ottawa Heart Institute’s Berkman Library space is outdated. Budget constraints and tiny square footage leave little room for error. A needs assessment using user experience (UX) research methods was conducted from 2022 to 2023 to inform strategic decisions on updating and reorganizing furnishings to better support library patrons and their needs.

**Methods:**

Data was collected via an electronic survey, “guerilla” interviews, observations of library patrons, and a physical survey of communal spaces in the building. Resulting qualitative data were compiled and examined for common themes. Low fidelity mockups of furnishings and space arrangements were prototyped and presented to patrons for feedback.

**Results:**

Quiet was one of the most valued attributes of the library space and showed itself to be a unique quality of the library when compared to communal spaces within the hospital. Survey and interview responses consistently cited soft, comfortable furnishings as desirable additions. Observed behaviours support the continued need for desks with a deep surface area to accommodate multiple devices used in tandem. Flexible use of computer hardware, better access to power outlets, and adjustable lighting were identified as additional gaps.

**Discussion:**

Methods showcase light-weight space assessment strategies that are of particular interest to solo librarians or small library teams working in a hospital environment. Results identify library qualities that address institutional gaps and provide insight into the motivators, needs, and behaviours of hospital staff. Centering patron behaviours and preferences in the project’s methodology provides data to support decision-making for near term upgrades and long-term library policy.

## Introduction

The University of Ottawa Heart Institute (UOHI) is Canada’s largest heart health centre employing over 1,650 health professionals [1]. With its mission to lead in patient care, research, and education [2], UOHI is a clinical care facility with an internationally recognized research program that also provides academic teaching and training in partnership with the University of Ottawa (uOttawa). In this context UOHI and uOttawa have maintained a memorandum of agreement for the operation of a uOttawa satellite library in the UOHI facility dating back to 2009. The library is known as the Berkman Library.

Throughout this period of partnership, services at the library have kept pace with developments in health librarianship and medical research. Movement towards digital resources, the emergence of citation management software, increased emphasis on research impact and assessment, and growing demand for systematic review support are key examples of such developments.

The physical space of the library, however, has seen no major upgrades or developments in that same period. The library has access to a limited dollar amount allocated annually as part of its regular budget that can be used to finance new furnishings. Even with its tiny square footage of 570 square feet, library furniture is costly, and spending will need to be carefully planned as a multi phased project distributed over several budget cycles. A proposal to replace deteriorating library furnishings was submitted to management at uOttawa and UOHI and received support. This paper details the subsequent quality improvement project.

As a first step in planning furniture replacement, it was determined that a needs assessment using user experience (UX) research methods was required. A needs assessment is a “systematic set of procedures undertaken for the purpose of setting priorities and making decisions about program or organizational improvement and allocation of resources” [3]. A UX approach places the user at the centre of decision making. It uses methods that capture attitudinal and behavioural data, employs analysis and ideation processes that centre user feelings, thoughts, and behaviours, and has as its end, to inform changes to products or services that improve the quality of the experience of the end user [4,5].

Turning to the literature, behavioural and attitudinal data have been used to inform the development of library services and spaces in the academic library setting in the following studies: Priestner et al.’s study of user behaviours and preferences at the University of Cambridge Library utilized a range of UX techniques including observations, interviews, surveys, co design workshops, and LEGO play sessions. Study findings were used to develop recommendations to guide future space provision and design [6]. Eldermire reports on how an open source, observational data collection tool, a survey, and focus groups were used to document library space usage and better understand user preferences and pain points [7]. Hillman et al.’s seating sweeps, focus groups, and campus wide survey were used to develop an understanding of how a small academic library space was being used [8]. Schultz et al.’s surveys and semi structured interviews of first-generation university students demonstrate how these techniques can be used to better understand different populations’ use of academic library spaces and their sense of belonging and safety within those spaces [9].

Literature about space improvement in health sciences libraries was more difficult to find. A wide variety of nomenclature is used with many papers preferring terms such as “user studies” or “user led”, or simply “space planning” or “needs assessment” while reporting qualitative data collection methods and espousing similar quality improvement goals as UX work [10-14]. Campbell reports that health library workers feel their patrons face significant stress and urgency in their work, and gaining patron input without taking up too much of the patron’s time is felt to be a challenge [15]. This could translate into health library workers conducting UX work less frequently than their colleagues in other specialties. It is equally possible that health library workers simply publish less frequently about their quality improvement work.

Published examples of academic health sciences libraries using behavioural and attitudinal data collection methods to support space planning include: Aronoff et al.’s survey of medical students to understand their library use and needs as part of the planning a new medical library [10], Saragossi et al.’s analysis of student reported dissatisfaction in accreditation surveys to obtain administrative support and buy in for a physical space renewal [16], Norton et al.’s survey, focus groups, and onsite voting of floor and carpet samples to obtain user input for a library renovation [13], Prentice et al.’s floor sweeps to observe library space and furniture usage [14], and Bayley et al.’s use of seating sweeps, a survey, and a series of observations to support an operational review of physical space use and services [17].

Only two examples of hospital based library space planning were identified in the literature that gathered attitudinal or behavioural user data: in 1997 Crabtree et al. distributed a survey to non physician hospital employees to obtain a baseline of their library usage and solicit recommendations for library improvements [11], and in 2007 Karunanayake described the dissemination of a paper and electronic survey to hospital staff to gauge satisfaction and needs with space, services, and web resources [12].

The only space planning literature we were able to identify from a hospital library context detailed the loss or renegotiation of space [18-21], or described renovations conducted [22,23], but none reported gathering attitudinal or behavioural user data.

This article seeks to address this gap in the hospital library literature in two ways: First, we will provide an example of how information professionals working alone or as part of small team in a hospital library context can use UX methods for space assessment. Second, we will share what we learned while investigating the following questions in our own context:

1. What types of furnishings are desired by library patrons?
2. How should furnishings be organized?
3. What elements are currently lacking or could be improved upon to increase the comfort and enjoyment of the library space for library patrons?

## Methods

### Context

**Fig. 1:**
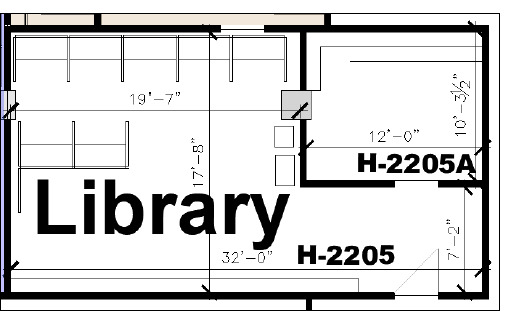
Berkman Library CAD (computer aided design)

**Fig. 2:**
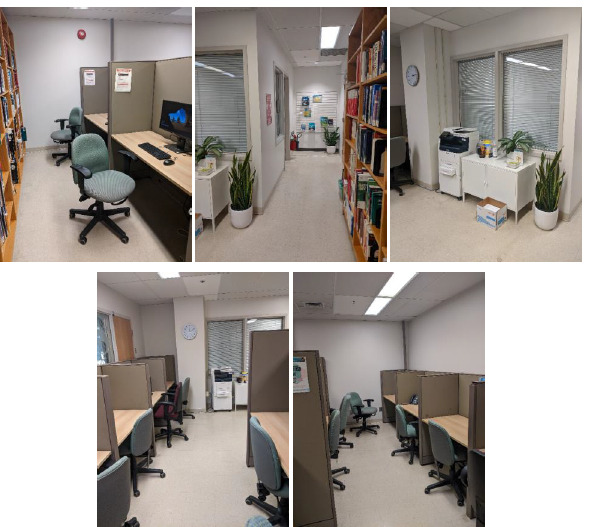
Collage of five images of the Berkman Library prior to assessment (2022)

The library is 570 square-feet and comprises of a librarian’s office, a wall of bookshelves, nine workspaces, a printer, and a cabinet (Figures 1 and 2).

Located on the second floor, the library is near various outpatient units, the rehabilitation centre, several research labs, and shared trainee spaces. A variety of patron groups access the second-floor space including nurses, physiotherapists, lab technicians, summer students, and staff physicians. The space is locked but remains accessible day or night to UOHI staff and trainees via their institutional security badge. Door count data collected by the security badge mechanism captures an annual total which unfortunately does little to provide any insight into space usage patterns. However, during weekday hours, the most frequently observed visitors include residents, clinical fellows, and post doctoral fellows.

### Project team members

The project team consisted of the UOHI librarian and the uOttawa user experience (UX) librarian. The UOHI librarian was the project stakeholder and initiator. As the sole staff member of the Berkman Library, the UOHI librarian is responsible for providing all onsite library services, collection development, marketing and promotion, and operations. The UOHI librarian conducted the data collection for the project (detailed later). The UX librarian led the project processes such as the design of the assessment methods, the analysis process, and the formulation of the findings.

### Data collection

The needs assessment consisted of five UX data collection methods. Each is described individually in detail, below:

1. An electronic survey
2. “Guerilla” interviews
3. Observations of library patrons
4. A physical survey of communal spaces in the UOHI building
5. A voting exercise on low fidelity mockups

These five data collection activities were small in scale and were completed over a series of months. They required no sophisticated set up or burdensome scheduling and could be carried out by the UOHI librarian within her existing workload. Data collection methods were chosen to be complementary, with the resulting data providing a more complete picture of the behaviours and priorities of people using the library than any single method. The project was undertaken for library improvement purposes; therefore, no ethics board review was required [24].

### Electronic survey

A short electronic survey was created using SurveyMonkey [25] and served to capture primarily qualitative data. The survey used open, closed, and multiple choice questions to capture information about the goals and priorities of people using the library (see Online Supplement, Appendix 1 for the survey instrument). The survey was distributed to all UOHI staff and trainees through an institutional email newsletter. It was additionally posted on the institutional intranet banner, advertised on flyers, posted in staff spaces, and promoted in person by the UOHI librarian. At the end of the survey, participants were invited to enter their email address for a chance to win 1 of 5, 10$ gift certificates to a coffee shop in the UOHI. Winners were randomly selected. The survey was open for 4 weeks (October 28 to December 1, 2022) and collected 29 responses.

Appendix 1

### “Guerilla” interviews and observations

To complement the survey responses, people in the library were interviewed and their workspace was observed. A “guerrilla” interview, so called by UX researchers, is an interview technique that engages people as participants who happen to be present and available on the spot. The technique does not “involve rigorous recruiting or screening processes or any kind of preparation by participants” [26] yet can produce useful research insights.

Signage was posted in the library to notify people that data gathering was taking place and to communicate the context and purpose. To ensure consistent data collection, a data-capture form was created (see Online Supplement, Appendix 2 for the data-capture form). Participants were asked if they were willing to participate before any data was captured. No identifying information was collected.

Appendix 2

In total, 12 observations and interviews were conducted, lasting no more than 5 minutes per participant. Participants answered the interview questions out loud while the UOHI librarian made notes about how the individual was using the workspace. Between 10 and 20 interviews is considered sufficient to provide workable data [4]. Therefore, once a point was reached where no new behaviours were observed, data collection was stopped. Sessions were conducted over a 4 week period (November 9 to December 5, 2022).

### Survey of communal spaces

To better contextualize the library space within the UOHI building, a physical survey of communal staff spaces located elsewhere in the hospital building was undertaken. As with the guerilla interviews and observations, a data collection form with standardized fields was created to ensure consistent data capture. Two thirds of this data collection form was an empty box (see Online Supplement, Appendix 3 for the data collection form). Here the UOHI librarian sketched the furniture and other elements observed during the space surveys. In total, 11 communal spaces were surveyed. Not all staff spaces were surveyed as some spaces are restricted to specific groups of staff and only those spaces that the UOHI librarian was aware of, and could gain access to via another staff member, were surveyed.

Appendix 3

### Mockups

As a final step, insights from the thematic analysis (described in the data analysis section) were translated into low-fidelity mockups of the library space. Using a computer aided design (CAD) file of the library to provide rough scale (Figure 1), Microsoft PowerPoint [27] was used to produce 4 low fidelity representations of different furniture choices and seating arrangements (see Online Supplement, Appendix 4 for the mockups).

Appendix 4

8 respondents who had completed the electronic survey and had expressed willingness to provide more feedback were sent the mockups via email. In the email they were asked to vote for their preferred mockup and explain their preference. 4 votes and 4 explanations were received.

**Fig. 3:**
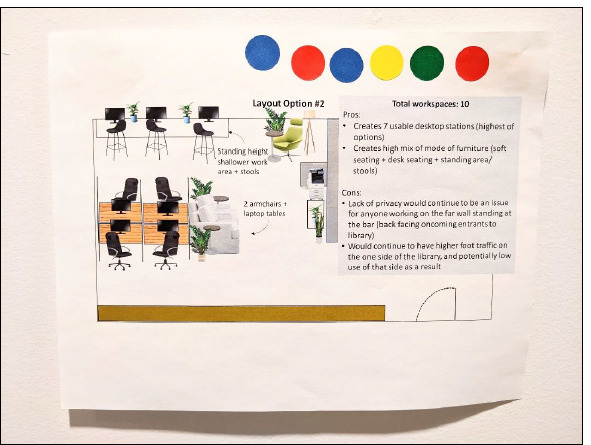
The mockup that received the highest number of votes

Mockups were also printed out and posted in the library for 4 weeks (March 24 to April 25, 2023). People visiting the library were invited to use coloured stickers to vote for the mockup they liked best. After several days without responses a “dummy vote” sticker was placed on one of the mockups to show library patrons the intended purpose of the stickers and allay perceived pressure of being the first one to vote. After this, 5 true votes were received (Figure 3).

### Data analysis

The data resulting from the UX data gathering activities was saved in a shared, cloud based file storage system. The electronic survey data was exported from the SurveyMonkey tool to Microsoft Excel [28] and the data collection forms were digitized. Video conferencing software was used for discussions and Miro [29], a virtual whiteboard that allows simultaneous collaboration, was used for data organization, analysis, and ideation processes.

### Affinity mapping

The authors used a technique called “affinity mapping”, where sticky notes are used to organize research findings and identify themes [30]. For this project, the project data was transferred to virtual sticky notes in Miro and then sorted into categories. When a narrative response contained more than one idea, each idea received its own virtual sticky note. Icons were used to represent items that were present during the observations (such as laptops, cell phones, etc.).

In preparation for the affinity mapping activity, the UX librarian transferred the research data onto virtual sticky notes, producing 142 sticky notes and 53 virtual icons. The colour of the sticky notes was arbitrary.

**Fig. 4:**
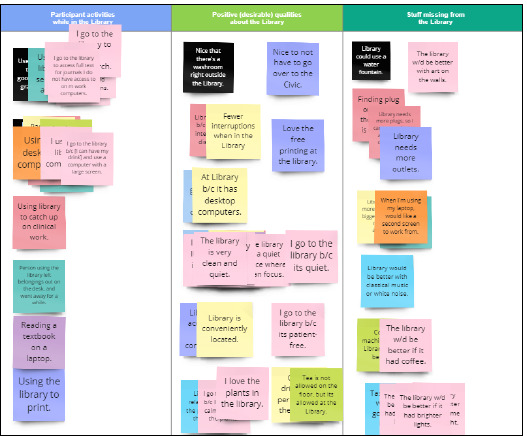
A snapshot of virtual sticky notes organized on a Miro board

While connected in a conference call and working simultaneously, the authors each took a virtual pile of sticky notes and icons and began to organize them on a shared Miro board. Where an idea was repeated, sticky notes or icons were piled together. Where ideas contrasted, they were piled side by side (Figure 4).

Once affinity mapping was complete, the sticky notes were spread out so each note could be read separately but remained within the context of other ideas on the same topic. By the end of this process, the authors were both intimately acquainted with the data.

**Fig. 5:**
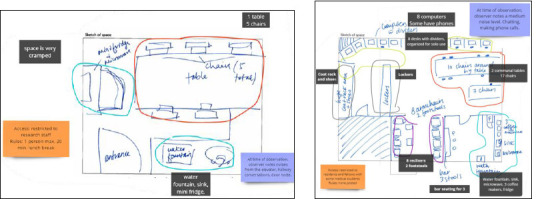
Physical surveys of two communal areas, marked up in Miro. The space on the left was restricted to research staff. The space on the right was restricted to clinical staff

Unlike the other data, the physical surveys did not lend themselves to affinity mapping. Instead, the UX librarian, who had not conducted the physical surveys, reviewed the collected survey data and independently highlighted common and contrasting elements using coloured pen and label functions in Miro (Figure 5). The authors then came together to discuss their thoughts and reflections.

### Thematic analysis

**Fig. 6:**
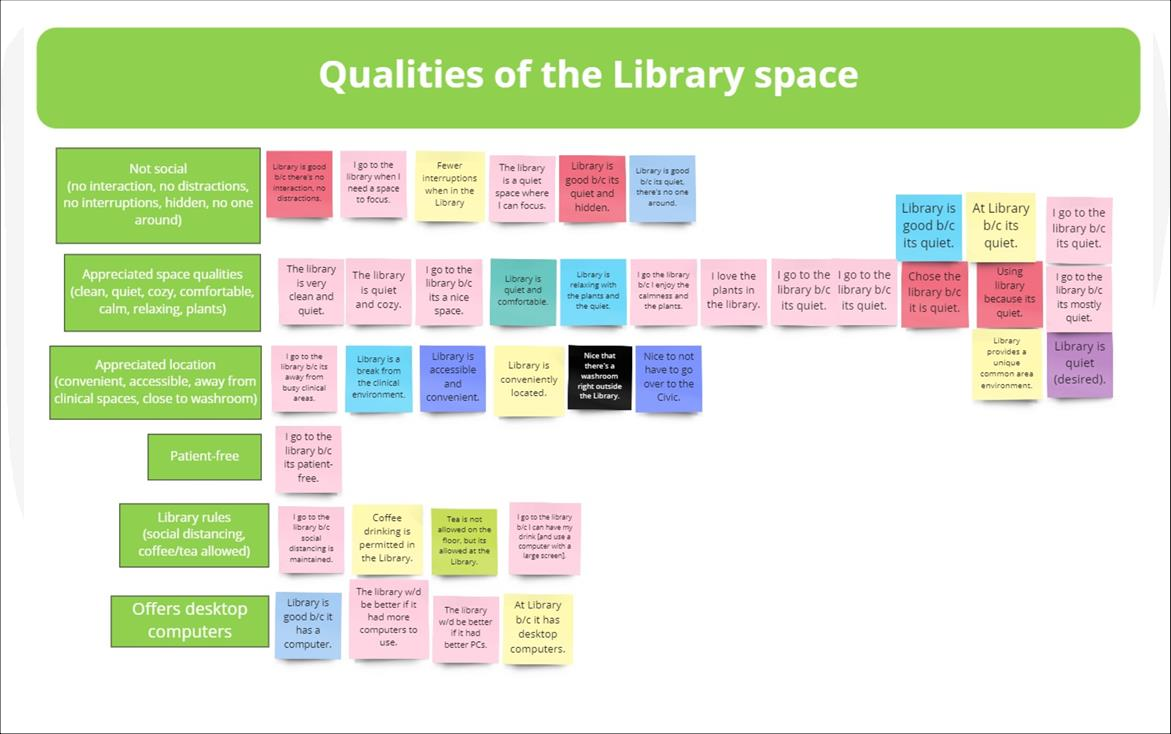
Snapshot of the thematic analysis process on a Miro board

The next step was to undertake a thematic analysis, “a systematic method of breaking down and organizing rich data from qualitative research by tagging individual observations and quotations with codes, to facilitate the discovery of significant themes” [31]. A single word or a short phrase was assigned to each pile of sticky notes or icons to describe what that pile was about (Figure 6).

## Results

The data analysis produced themes that helped the authors understand the behaviours, preferences, and values of people using the library, as presented in their own words or through their actions. These themes replaced anecdotes and complemented the professional experience of the UOHI librarian. Table 1 summarizes the themes drawn from the needs assessment data.

**Table 1:** Summary of themes drawn from the needs assessment data

**Participants were observed in tde library witd…**	Secondary devices (mobile phones, laptops, tablets) Tools (papers, flashcards) Food, drinks Bags, clotding, and masks
**Participants told us they go to the library to…**	Use a desktop computer Access an individual workspace Print materials Meet with the librarian
**Participants told us they liked the library because…**	It is quiet It is clean It allows freedom to consume drinks It is comfortable, relaxing, and has plants It is non social (“no interaction”, “no distraction”, a “hidden” space) and is patient free It provides access to desktop computers It is well located (proximity to washrooms, distant from clinical areas, and “convenient”)
**Participants told us that the following elements would improve their experience of the library…**	Control over workspace lighting Adaptable workspaces for different bodies and working preferences Windows or artwork Easier access to electrical outlets Larger computer monitors Assurance of space availability before arrival Comfortable, relaxed furniture Group spaces Rooms for individual use
**When seated in the library, participants were observed to…**	Avoid sitting side by side or back to back Prefer sitting against the wall
**Other workspaces in the UOHI building were described by participants as…**	Being social and busy Not providing access to research materials, computers, or workspaces Not being comfortable
**Other workspaces in the UOHI building were observed to…**	Provide space and facilities for eating Provide few desktop computers, and of these, none gave the user privacy Provide few printers or plants Provide some spaces with furniture configured for social interaction and others for individual use Provide only open concept spaces Provide clinical staff with spaces that felt spacious with natural light and soft furnishings Provide research staff with spaces that felt cramped with no naturallight and hard furnishings Provide spaces that were restricted to a subpopulation of staff. The exceptions were: the cafeteria, a staff lunchroom, and the library.

The data analysis also uncovered potential actions, i.e. ways the library could respond to the research findings. These potential actions are the result of an additional step after the data analysis, where brainstorming is used to come up with ways to respond to the findings of the research. To be useful, these actions needed to be specific, therefore “improve” or “make better” statements which Priestner cautions can result in “empty wish[es]” [4], were avoided. Table 2 summarizes potential actions resulting from this idea generation work.

**Table 2:** Potential actions resulting from idea generation work

**Equipment**
Continue to prioritize desktop computers Provide dual or larger screens Provide HDMI cables to plug in personal devices to library screens Add furniture with integrated power Retain the printer in the library Add task lighting
**Furnishings**
Add variety to the workspaces offered with relaxed and standing options Provide footstools Maintain some private workspaces and some large sized individual workspaces Purchase furniture with integrated shelving, side tables, and/or hooks to maximize availableworkspace for people Orient furniture to discourage social interactions
**Atmosphere**
Prioritize quiet as a defining feature of the library’s space Consider removal of the staff use phone in the library Monitor impact of food and drink consumption on the cleanliness of the space Increase the number of plants (bring the outdoors in, increase privacy, elevate mood) Add elements (art, plants, etc.) people can use to change the focus of the eyes Choose calming textures and colours when selecting new furniture Introduce sound absorbing materials
**Other**
Investigate the cost benefit of an occupancy or reservation system

## Discussion

### Comparing the library to other UOHI communal spaces

Results of the needs assessment suggested some of the most appreciated qualities of the library space were also what set it apart from other communal spaces in the UOHI. As seen through the communal space surveys, the library appeared to be one of the few employee-only spaces open to all UOHI staff and trainees, equally convenient for both research and clinical staff. In comparison to communal spaces for research staff, the library appeared to be one of the few comfortable spaces research staff could access. For clinical staff, the library was one of the few quiet, non social spaces they could access.

Gaining a better understanding of communal staff spaces located elsewhere in the hospital and reflecting on the differences between those spaces and the Berkman Library, was eye opening. It was remarkably impactful for the authors to see comments valuing library supplied desktop computers and then to see those comments reinforced by observing how few desktop computers were available to clinical and research staff in communal spaces outside the library. Combined with anecdotal reports that many of the hospital’s staff (e.g., residents, clinical fellows, floor nurses) are not provided with workspaces, this information deeply alters the UOHI librarian’s understanding of the value of providing computer access in the current context of the hospital. It has been a long-held assumption that the library’s desktop computers should be decommissioned in the long term in favour of a bring-your-own-laptop approach. Yet this needs assessment shows a bring-your-own-device approach would have a negative impact on a current group of library patrons. Striking a balance between continuing to supply some computers but also enabling peripheral connections, such as by supplying HDMI cables to connect personal laptops to monitors, will be key to moving forward.

### The value of quiet

Though some of the findings from the needs assessment appear obvious, they are important because they confirm the UOHI librarian’s experience and explain the value of certain library tenets within the context of the UOHI. Appreciation for quiet, for example, was a predominant message in participant feedback. Day to day, there are few complaints about noise and for the most part the library *is* quiet. Yet recently the UOHI librarian has observed an increase in the number of requests to use the library to make phone and video calls. Participant feedback from the needs assessment suggests that library patrons see *quiet* as a rare commodity in the context of the hospital. This insight into how unique quiet is within the institution, particularly when coupled with the level of privacy that the library offers, positions the UOHI librarian to make evidence-informed decisions to protect the library’s unique character through policy, furniture, and space design. Indeed, the necessity for a *quiet* library space within the UOHI is no longer a function of tradition but can now be a deliberate choice.

### How people work

This needs assessment set out to gather information about the types of furnishings library patrons desire as well as how those furnishings should be organized. While the library’s existing carrels provide privacy, ample worksurface, and a significant number of seats, it is a singular vision of what working is. The assessment findings invited the authors to re-examine this vision of what *working* is in terms of furniture choices but also the assumption that more seats translate to higher occupancy. In observations, people left the chair between them and the next person vacant if they had a choice. Sitting back to back was also avoided. By adapting the relationships between the furniture, an element explored in Cambridge’s Protolib Project [6], the library might be able to achieve equal or higher overall occupancy with fewer total chairs.

On days where she conducted Guerilla interviews, the UOHI librarian noted when patrons arrived and when they left, recording a rough measure of the total time patrons spent in the library. The collection strategy for this data, however, quickly showed itself to be problematic. In some instances, the UOHI librarian ended her day before library patrons departed while in others, patrons were already comfortably installed when she arrived. That said, this data was able to show the authors that most patrons were spending one or more hours when they decided to work in the library. Therefore, the library needs to ensure furniture is suitable for longer term occupancy.

Feedback suggested people also have different views on what they need from workspaces yet the library currently only offers chairs at cubicles. Nearly one third of the improvement related comments spoke directly to furniture, offering terms such as “lounge”, “comfy”, “comfortable”, and “casual”. Participants also signaled a desire for control over their workspace lighting, the ability to adapt the workspace for their needs, and the option to choose between different types of workspaces. The library could explore adding more variety to the working spaces offered by introducing standing, soft, relaxed, or adaptable workspaces.

Facilitating personal device use could be another area where the library could offer more variety in work-style options. Improving access to power outlets for personal devices, providing connector cables and more dual monitors to facilitate a build-your-own workstation approach would allow library patrons to transform or enhance the functionality of their personal devices or use personal devices alongside the library’s desktop computers.

### Convergent and divergent responses

In analyzing the data, the authors observed both convergent and divergent responses from participants. Some participants wanted library space dedicated to individual activities while others wanted spaces designed for group use. Study cubicles were prized by some while others wanted armchairs.

As one of this assessment’s objectives was to better understand what was lacking from the library’s space from the perspective of patrons, the authors needed to examine both the ideas in the data and participant motivations to make decisions about next steps. *Quiet* was a dominant idea, but the reasons for wanting quiet space differed. For some participants, quiet helped them focus while for others it was better for making calls. Likewise, library workspaces were important but the reasons for why they were important differed. Some participants said they had no dedicated workspace of their own, so they used the library. For others, they had a dedicated workspace, but it lacked equipment or was in an atmosphere they felt interfered with their ability to work, such as in a social or high traffic area.

A key take away from this needs assessment was the importance of looking beyond what participants say they want to forming an understanding of *why* library patrons want certain elements. In understanding the why, we get closer to understanding the library’s value proposition. In other words, it is by working to understand the why that we begin to develop a more nuanced understanding of the reasons library patrons choose the library over alternatives. This knowledge gives library staff confidence and agency when advocating for library spaces or adapting services.

### Risk and decision inertia

On a practical level, the prospect of discarding and purchasing new furniture seemed daunting and risky prior to this assessment. With the impact of inflation on purchasing power and material shortages related to the COVID-19 pandemic, some furniture has been priced out of the library’s reach altogether. It is a sobering reality that a single new piece of furniture could consume nearly the entirety of the year’s available budget. Therefore, creating a plan centered on patron feedback helps break out of indecision inertia.

### Advocacy

Politically, the findings from this needs assessment give the library strong evidence of the unique role the library space plays within the context of the larger institution. Libraries and library services often suffer during economic downturns [32-38], and hospital libraries seem particularly vulnerable to cuts, closures, consolidations or mergers, and employee attrition [18-21,39-44 ]. Given that hospital libraries tend to also have smaller teams or in some cases run by a single staff member, a template for engaging in authentic user research and meaningful action while also not overburdening library staff ensures the library is listening and being responsive to the needs of its patrons while providing the library with ready justification and evidence should advocacy be required.

### Strengths and weaknesses

Even though this needs assessment was carried out by a small (two person!) team, having more than one perspective when examining the data richness to discussions, helped safeguard against some individual biases, and brought more diversity to data interpretation. Using a variety of research methods built a fuller user experience story than any single method could alone. The selected research methods were lightweight for the UOHI librarian and not overly burdensome to library patrons. Investing time and effort into the processes of data analysis and idea generation increased the authors’ understanding of patron needs and the library’s potential response.

Patient care providers and researchers are busy people. Despite the variety of data collection activities conducted, the needs assessment data may not include the perspectives of those who do not use the library or use it for less traditional purposes, are infrequent library visitors, or were unavailable during the data collection period. Some respondents may have contributed to more than one data collection activity, the dummy vote placed on the library mockups may have biased some respondents, and not all communal staff spaces elsewhere in the hospital were surveyed.

Further, the survey and the mockup activity saw low response rates which could signal a potential risk to data quality. This risk was managed, however, by the choice to use a variety of data collection methods (a survey, observations and interviews, and communal space surveys). The data from the different data collection methods was triangulated together to avoid reliance on any single data source. For the mockups, the intention was to hear from library patrons but not necessarily for the preferred mockup to be the final iteration. Indeed, we expect the design to continue to evolve while the mockup will serve primarily as concrete representation of the direction the library is headed in.

### Future directions

This needs assessment has enabled the authors to observe patrons at work, visit alternative workspaces in the hospital, hear how patrons say they wish to work in the library, and become aware of some patron motivations for choosing the library over alternatives. The collected data of observed behaviours and library patron beliefs and values has been transformed into potential actions and a visual representation that communicates a desired future state.

As for next steps, the UOHI librarian will use the preferred mockup and the list of potential actions to develop a plan to present to management which will communicate the space design vision and the changes that are needed. Steps of the plan will be prioritized and then plotted on a timeline. Consideration will be needed to minimize disruptions for library patrons and to manage spending given the library’s small budget for new furnishings each year. Likely this will result in a staggered approach to furniture acquisition and upgrades that take place over several budget cycles. The UX findings from this project are context specific and cannot be generalized. Yet the lightweight data collection methods and data analysis processes presented in this paper offer a blueprint that other solo library workers or small hospital library teams may be interested in replicating within their own contexts.

## Data Availability

The data collection tools are provided in the manuscript appendices. Given that this was a quality improvement project and participants did not give written consent for their data to be shared, the data for this needs assessment is not publicly available.
