# Supplementary material for: Tiny? Make it mighty! Maximizing a limited-budget upgrade of a pint-sized hospital library using UX methods
Source: J Can Health Libr Assoc. 2024 Dec 1;45(3):161–75. doi: 10.29173/jchla29774 (PMC11881648; doi:10.29173/jchla29774)
Supplement: Supplementary file 1 [file JCHLA-45-161-s001.pdf]

Appendix 1

Electronic survey

The Berkman Library (H-2205) is planning a facelift! Help us get it right. Take our one-minute Library Facelift Survey! Your experience using the library space to focus, take a break, or do what you do while you're there, will help us make choices that reflect your needs.

As a thank you, you'll be entered in a draw to win one of five $10 Tim Hortons gift cards.

No time for surveys? No problem: send your thoughts to [insert UOHI Librarian email address].

1. I go to the library to…
    a. Use the computers
    b. Chill ('nuff said, we all need to take breaks!)
    c. Check out books
    d. See the librarian (hellooo!)
    e. Other: (please specify)

2. I go to the library because…

3. The library space would be more useful or comfortable for me if …

4. My role at the UOHI is:

5. I am interested in talking more about my experience using the library space.
   Yes, I am interested. Here is my email address:
   No, thanks!

6. Join the draw to win one of five $10 Tim Hortons gift cards (this email will be kept separate from your responses to preserve anonymity).

Thank you for taking the time to fill out this survey. See you at the Library!
