# Supplementary material for: Tiny? Make it mighty! Maximizing a limited-budget upgrade of a pint-sized hospital library using UX methods
Source: J Can Health Libr Assoc. 2024 Dec 1;45(3):161–75. doi: 10.29173/jchla29774 (PMC11881648; doi:10.29173/jchla29774)
Supplement: Supplementary file 2 [file JCHLA-45-161-s002.pdf]

Appendix 2

Data collection tool for the in-person observations and interviews

Date:

Time of day:

Observations

Are they using the desktop computer? Y/N

What personal device(s) are visible on their workspace? (list)

-
-
-

What other items are on their workspace? (list)

-
-
-

What items did they bring and are on the floor, on the chair, under the table? (list)

-
-
-

How long do they stay, approximately? (circle)

0-0.5 hrs   0.5-1hr   1-3hrs   3+hrs

What seat do they choose?

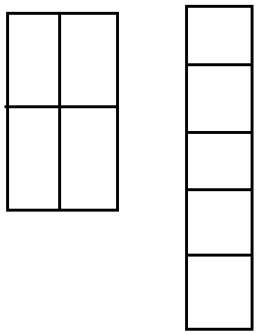

Interview questions:

Hi, sorry to interrupt!

I'm Sarah, the Librarian. I'm observing how people are using the library space as part of an improvement project I'm working on. Can I ask you 3 quick questions?

1. What activities are doing while you're here in the library today?

2. What made you choose the library over another space elsewhere?

3. What's missing from this space that would make it better for you?
