# Supplementary material for: Tiny? Make it mighty! Maximizing a limited-budget upgrade of a pint-sized hospital library using UX methods
Source: J Can Health Libr Assoc. 2024 Dec 1;45(3):161–75. doi: 10.29173/jchla29774 (PMC11881648; doi:10.29173/jchla29774)
Supplement: Supplementary file 3 [file JCHLA-45-161-s003.pdf]

Appendix 3

Data collection tool for communal space observations.

Date:

Location:

Sketch of space

Observations:

-
-
-
