# Supplementary figures and images for: Tiny? Make it mighty! Maximizing a limited-budget upgrade of a pint-sized hospital library using UX methods
Source: J Can Health Libr Assoc. 2024 Dec 1;45(3):161–75. doi: 10.29173/jchla29774 (PMC11881648; doi:10.29173/jchla29774)

## Appendix 4

*Mockups of Berkman Library spaces and furniture.*

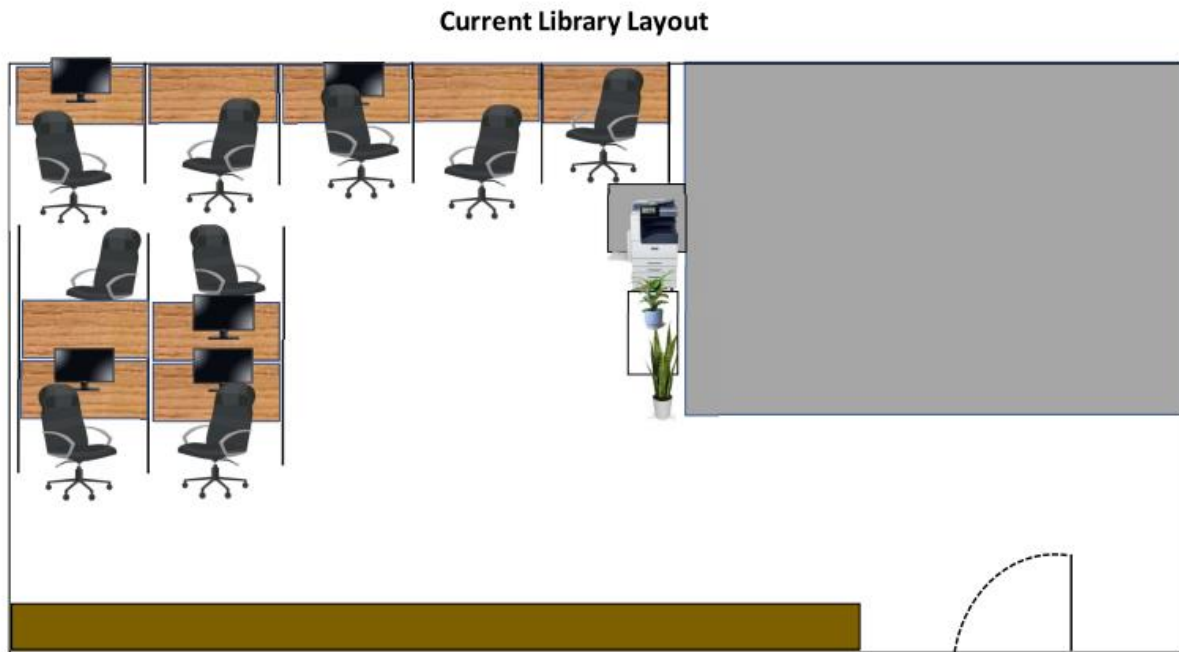

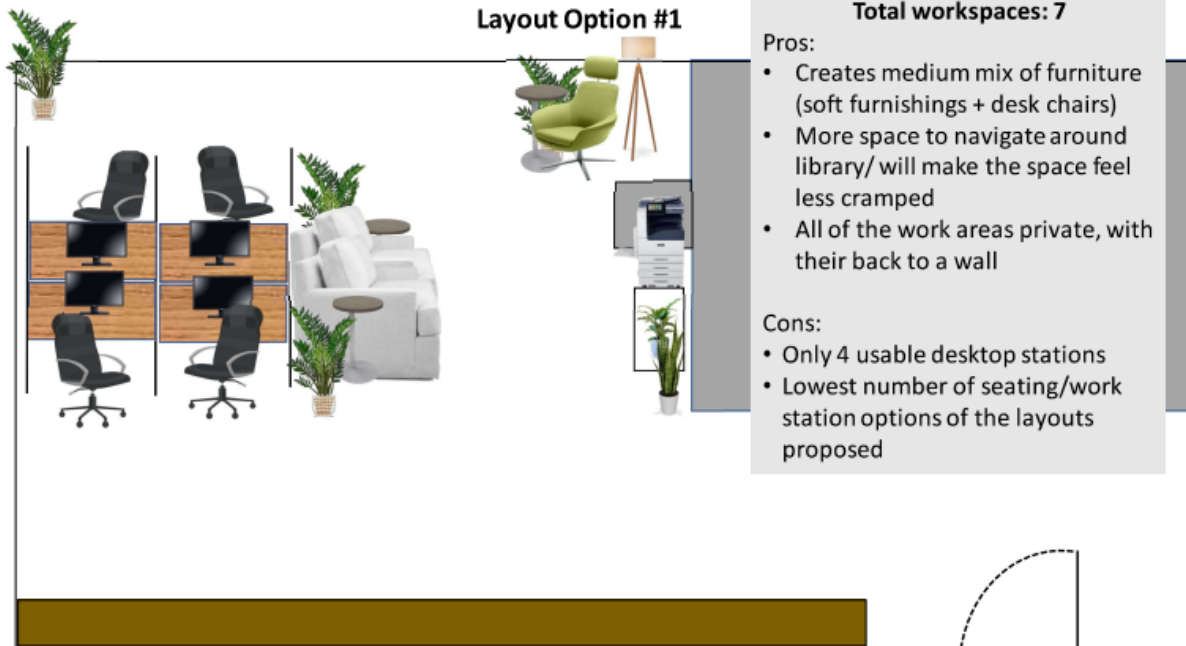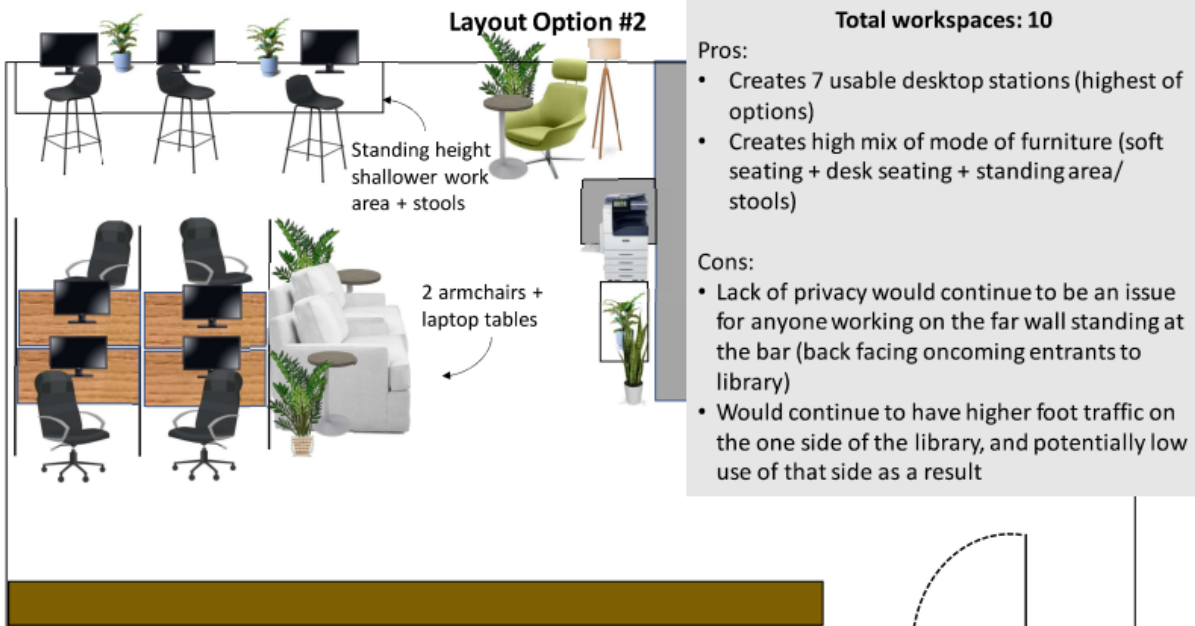

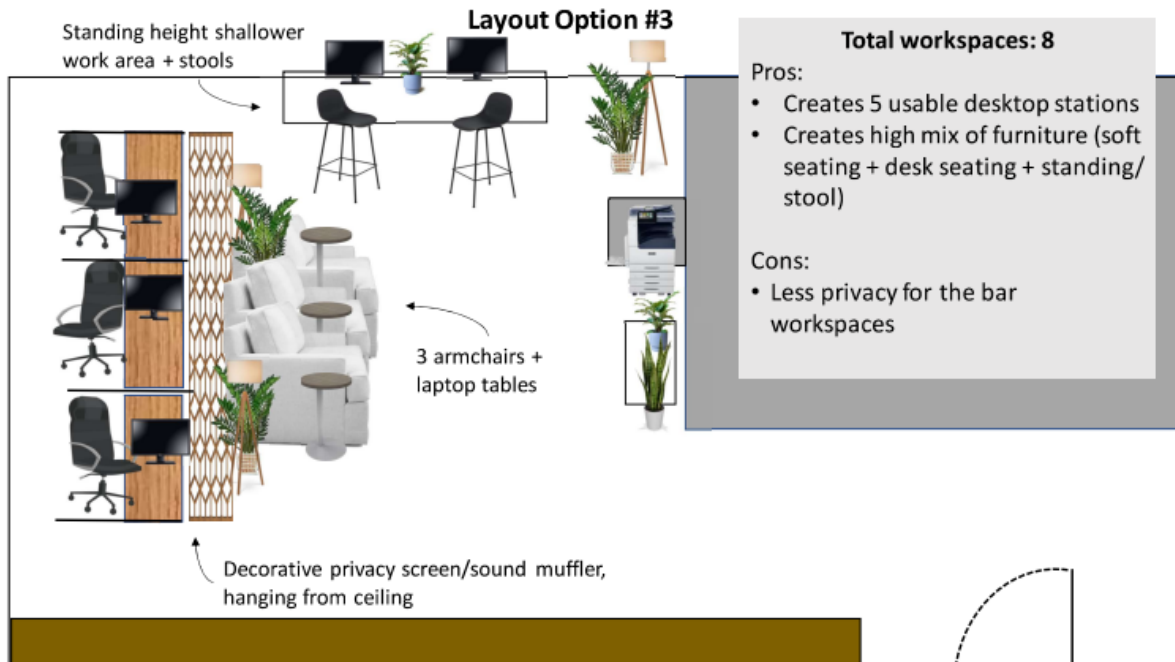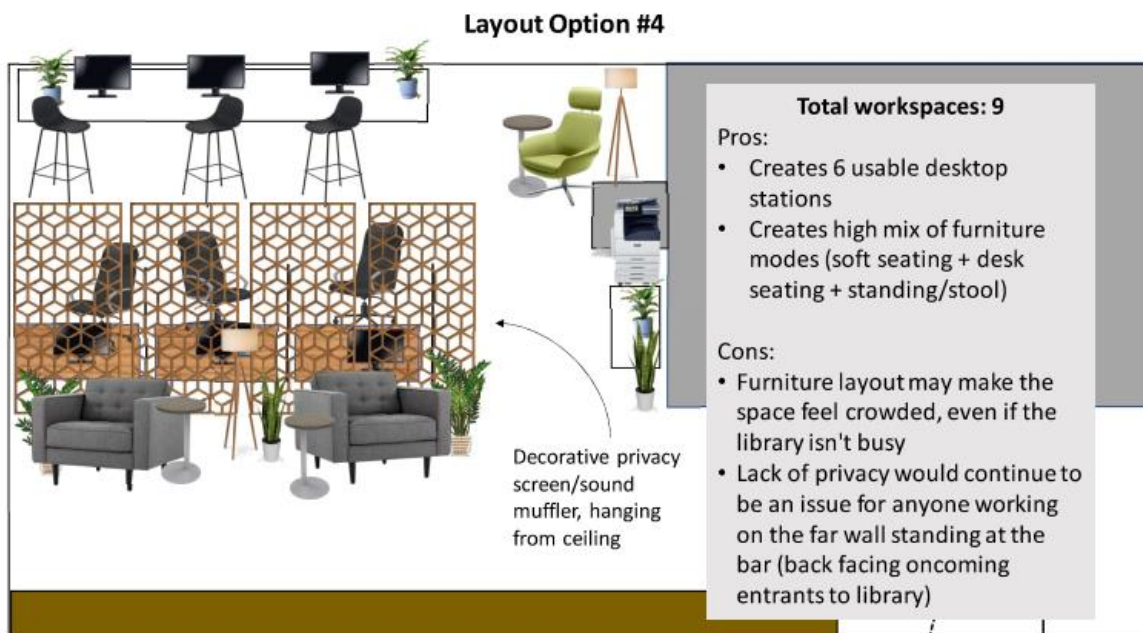

Supplement: Supplementary file 4 [file JCHLA-45-161-s004.pdf]
